# Pathogenic relationship between phenotypes of ARPKD and novel compound heterozygous mutations of *PKHD1*

**DOI:** 10.3389/fgene.2024.1429336

**Published:** 2024-07-02

**Authors:** Xinrong Zhang, Jiebin Wu, Jianteng Zhou, Jie Liang, Yu Han, Yunmeng Qi, Tao Zhu, Dejian Yuan, Zuobin Zhu, Jingfang Zhai

**Affiliations:** ^1^ Xuzhou Central Hospital, Xuzhou Clinical College of Xuzhou Medical University, Xuzhou, China; ^2^ Xuzhou Engineering Research Center of Medical Genetics and Transformation, Key Laboratory of Genetic Foundation and Clinical Application, Department of Genetics, Xuzhou Medical University, Xuzhou, Jiangsu, China; ^3^ Department of Prenatal Diagnosis Medical Center, Xuzhou Central Hospital, Xuzhou, Jiangsu, China; ^4^ Key Laboratory of Brain Diseases Bioinformation of Xuzhou Medical University, Xuzhou, Jiangsu, China; ^5^ Department of Medical Genetics, Liuzhou Municipal Maternity and Child Healthcare Hospital, Liuzhou, China

**Keywords:** autosomal recessive polycystic kidney disease, PKHD1, minigene splicing assay, pathogenic mechanism, whole exome sequencing (WES)

## Abstract

**Background:**

To investigate whether the novel mutation of *PKHD1* could cause polycystic kidney disease by affecting splicing with a recessive inheritance pattern.

**Methods:**

A nonconsanguineous Chinese couple with two recurrent pregnancies showed fetal enlarged echogenic polycystic kidney and oligoamnios were recruited. Pedigree WES, minigene splicing assay experiment and following bioinformatics analysis were performed to verify the effects, and inheritance pattern of diseasing-causing mutations.

**Results:**

WES revealed that both fetuses were identified as carrying the same novel mutation c.3592_3628 + 45del, p.? and c.11207 T>C, p.(Ile3736Thr) in the *PKHD1* gene (NM_138694.4), which inherited from the father and mother respectively. Both bioinformatic method prediction and minigene splicing assay experience results supported the mutation c.3592_3628 + 45del, p.? affects the splicing of the *PKHD1* transcript, resulting in exon 31 skipping. Another missense mutation c.11207 T>C, p.(Ile3736Thr) has a low frequency in populations and is predicted to be deleterious by bioinformatic methods.

**Conclusion:**

These findings provide a direct clinical and functional evidence that the truncating mutations of the *PKHD1* gene could lead to more severe phenotypes, and cause ARPKD as a homozygous or compound heterozygous pattern. Our study broadens the variant spectrum of the *PKHD1* gene and provides a basis for genetic counseling and diagnosis of ARPKD.

## Introduction

Autosomal recessive polycystic kidney disease (ARPKD) is primarily a hereditary disorder characterized by multiple cysts in both kidneys and liver, with an estimated incidence of 1 in 20,000 births (Bergmann, 2017). The disease is linked to a protein called Fibrocystin/Polyductin (FPC) encoded by the *PKHD1* ciliary IPT domain containing fibrocystin/polyductin (*PKHD1*) [OMIM#606702] gene on chromosome 6p12.2 (Melchionda et al., 2016), which plays a vital role in maintaining the structural integrity of kidney and liver. Therefore, the presence of *PKHD1* mutation or reduced expression of DAZ interacting zinc finger protein 1 (DZIP1L) may account for the development of cystic phenotypes in both the kidney and liver, as well as functional abnormalities through influencing cellular proliferation, secretion, apoptosis, and terminal differentiation (Bergmann, 2017; Ziegler et al., 2020). The majority of patients with ARPKD typically present during the perinatal or childhood period, while a minority may present in adulthood. The severity of ARPKD symptoms varies depending on the age of onset, with the earlier onset associated with more severe manifestations (Bergmann, 2017; Cordido, Vizoso-Gonzalez, and Garcia-Gonzalez, 2021).

Considering as a recessive inheritance pattern of ARPKD, most patients carry compound heterozygous mutations with a more severe phenotype when both mutations are truncating (Benz and Hartung, 2021; Burgmaier et al., 2021; Cordido, Vizoso-Gonzalez, and Garcia-Gonzalez, 2021). Carriers with heterozygous *PKHD1* mutations are commonly thought not to develop cystic kidney and liver disease. However, it has been shown that heterozygous carriers of *PKHD1* mutations are at risk of developing cystic liver and kidney abnormalities (Gunay-Aygun et al., 2011). And enrichment of these mutations in *PKHD1* gene was observed on a genome-wide basis among a cohort of 102 unrelated patients with dominantly inherited isolated polycystic liver disease (30% had kidney cysts) (Besse et al., 2017). Furthermore, some experiments in mouse models have revealed that heterozygous truncating mutations of the *PKHD1* gene can cause cystic liver or kidney disease (Shan et al., 2019). Therefore, the inheritance pattern of ARPKD is so elusive that it is difficult for clinicians to provide accurate counseling for patients with *PKHD1* gene mutations of unknown clinical significance, especially for the families wishing to have a healthy offspring during pregnancy (Bergmann, 2017). As a result, it is important to clarify the pathogenicity of genotypes to assess the inheritance patterns and clinical phenotypes. However, many studies have not been further explored to confirm whether mutant sites have an effect on *PKHD1* gene function.

In this study, we reported a nonconsanguineous Chinese couple with two recurrent pregnancies exhibiting fetal enlarged echogenic polycystic kidney and oligoamnios in ultrasound. The same novel compound heterozygous *PKHD1* variants were detected in both of the fetuses inherited from the parents by whole exome sequencing (WES). Additionally, a minigene experiment was conducted to investigate the potential impact of this mutation on splicing. Here, we aim to explore the relationship between pathogenic genotypes and phenotypes of ARPKD.

## Materials and methods

### Clinical data

A 29-year-old healthy woman, gravida 2 and para 1, wanted a healthy baby in her second pregnancy and was referred to Xuzhou Central Hospital at 19+1 weeks in July 2021. The fetal structure was normal in ultrasound. The couple were nonconsanguineous and denied a history of high blood pressure, hereditary diseases, or drug usage. The pregnancy woman once underwent an induced labor procedure 2 years ago due to fetal polycystic kidney. And pedigree WES of parental peripheral blood and umbilical cord blood at 32-week gestation revealed novel compound heterozygous variants in *PKHD1* unreported previously. She accepted amniocentesis for prenatal genetic diagnosis. Both the karyotyping and copy number variation sequencing (CNV-seq) results were normal. However, Sanger sequencing confirmed that the fetus owned the same *PKHD1* mutations as the first fetus. The following prenatal ultrasound at 22-week gestation showed fetal bilateral normal-sized kidneys with enhanced renal cortexes. After the following 1 week, the similar change above and decreasing amniotic fluid (amniotic fluid index 7.5 cm) were presented in ultrasound of Nanjing Maternal and Child Healthcare Hospital. Oligohydramnios and bilateral enlarged echogenic polycystic kidney occurred at 26-week gestation (amniotic fluid index 33 mm). After being fully informed of the prognosis of the fetus, the couple terminated the pregnancy at 26 + 5 weeks of gestation. Subsequently, fetal autopsies were performed. This study was approved by the Ethics Committee of Xuzhou Central Hospital (XZXY-LJ-20190210-037), and written consent was obtained from the couple for publication.

## Methods

### Ultrasound examination

Prenatal ultrasound examination was conducted by ultrasound physicians using a color Doppler ultrasound instrument (GE Voluson E8, American) in accordance with the guidelines of the International Society of Ultrasound in Obstetrics and Gynecology (ISUOG). Fetal samples were obtained by ultrasound-guided amniocentesis or cordocentesis.

### Cytogenetic examination

Amniotic fluid or umbilical cord blood were cultured, harvested, prepared, G-banded, and analyzed on metaphases following standard procedure. The following karyotyping reports were interpreted according to the International System for Human Cytogenetic Nomenclature (ISCN 2016/ISCN 2020).

### CNV-seq detection

Uncultured genomic DNA was extracted from the samples using the QIAamp DNA Blood Mini kit (Qiagen, Germany), according to the manufacturer’s steps. The CNV-seq process was completed by the steps as described (Liang et al., 2014) through the following process: DNA fragmentation of each sample and libraries’ construction including end filling, adapter ligation and polymerase chain reaction (PCR) amplification. Subsequently, the DNA libraries were massively sequenced in parallel on the NextSeq 500 platform (Illumina, San Diego, CA, United States) to generate approximately 5 million raw sequencing reads with 36-base pair (bp) genomic DNA sequences.

### Whole exome sequencing (WES)

Pedigree WES of the first fetus and his parents was performed to detect single nucleotide variants (SNVs) and smaller genomic insertions and deletions (indels). Genomic DNA was extracted, hybridized and enriched from fetal cord blood and parental peripheral blood using SureSelect Human All Exon kit v6 (Agilent Technologies) according to the manufacturer’s protocol. Paired-end sequencing was performed on the Illumina Nova seq6000/Hiseq Xten platform (Illumina, United States) to obtain the original data following to the GATK Best Practices recommendations to call variants (Van der Auwera et al., 2013). Briefly, sequences were aligned to the GRCh38 reference genome with BWA-MEM (Version 0.7.17), followed by the removal of PCR duplicates and base quality score recalibration with GATK (Version 4.3.0.0). And single nucleotide variations and small indels were identified and quality-filtered using GATK’s HaplotypeCaller. Afterwards, we performed hard-filtering for SNPs and INDELs with GATK’s VariantFiltration. Finally, the detected mutations were annotated using ANNOVAR (Wang et al., 2010).

Candidate mutation screening was performed step by step as described in Supplementary Figure S1. After filtration, the mutation c.3592_3628 + 45del, p? and c.11207 T > C, p.(Ile3736Thr) in the *PKHD1* gene (NM_138694.4) were identified as candidate mutations. All variants were analyzed according to the American College of Medical Genetics and Genomics (ACMG) guidelines (Richards et al., 2015).

### Minigene splicing assay experiment

The splicing effect of the mutation c.3592_3628 + 45del, p? in the *PKHD1* gene (NM_138694.4) was further verified by a minigene experiment. The assay was carried out to examine the cDNA region of the target gene according to the candidate variant. Part of the *PKHD1* gene (partial intron 30 (217 bp) exon 31 (68 bp)- and partial intron 31 (973 bp)) were split and constructed in pcMINI vector (Supplementary Table S1). The wild type (WT) and mutant plasmids were confirmed by Sanger sequencing, then plasmids were transfected into HEK293T and HeLa cells separately using classical restriction and ligation methods. Total RNA was extracted from cells cultured for 48 h with Trizol (RNAiso PLUS) (TaKaRa, Kusatsu, Japan) and reverse-transcribed with the Superscript III reverse transcriptase (HifairTM 1st Strand cDNA Synthesis SuperMix for qPCR (YEASEN, Shanghai, China)), and the resulting cDNA was PCR-amplified with pcMINI-F/pcMINI-R primers (Supplementary Table S2). The amplified products were analyzed on 2% agarose gel electrophoresis. When the PCR products were mixed, the target fragments were cloned into plasmids by TA cloning for Sanger sequencing. The splice form of the mutant type was analyzed by comparing the sequencing results of wild-type and mutant-type. To substantiate the conclusion drawn in this study, the assay was conducted repeatedly in pcMINI-N plasmids by using the same experimental method as above.

### Bioinformatics analysis

Several websites, including SpliceAI (https://spliceailookup.broadinstitute.org/), NetGene2 (https://services.healthtech.dtu.dk/services/NetGene2-2.42/) and NNSplice (http://www.fruitfly.org/seq_tools/splice.html) were used to predict the potential effect on splicing.

Genes that may cause polycystic kidney phenotype were used to analyze the protein-protein interaction using the STRING database (https://cn.string-db.org/). Those genes are *PKD1, PKD2, PKHD1, DZIP1L, PMM2, GANAB, HNF1β, TSC1, TSC2, VHL, TCF2, NPHP1, DNAJB11, SEC63, PRKCSH, COL4A1, OFD1, ANKS6, NPHP2, NPHP3, NPHP9, DNAJB11, ALG8, ALG9, PRKCSH, SEC63, SEC61B, LRP5, SEC61A1, LRP5,* respectively. Cytoscape software was used to draw protein-protein interaction networks.

## Results

### Clinical features

**FIGURE 1:**
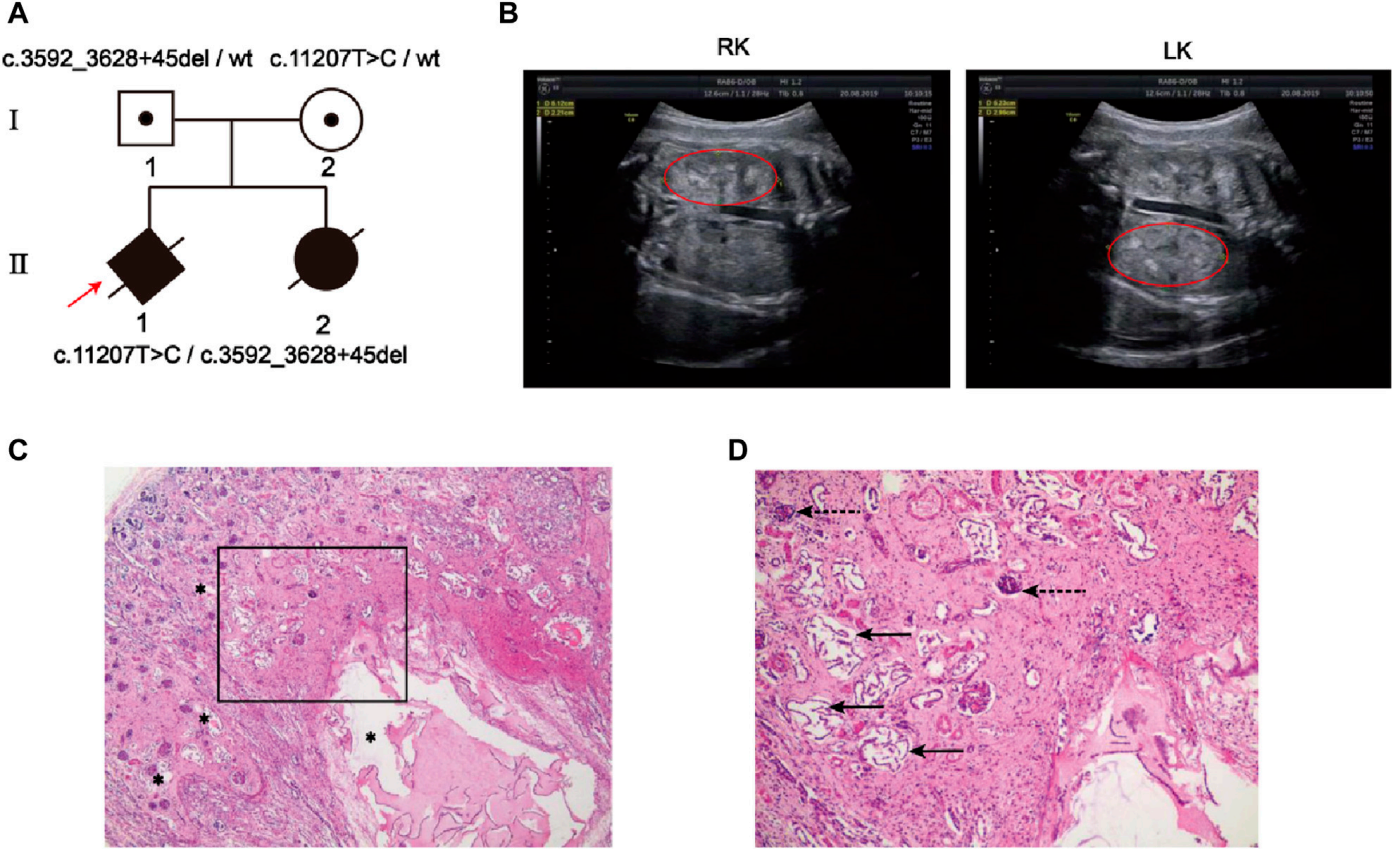
Clinical features of the family with autosomal recessive polycystic kidney disease. **(A)** Two members in this pedigree were diagnosed with ARPKD. Rotating solid square with an arrow indicates the proband (II-1); black spots, carriers; black solid shape, ARPKD patients; slashes, induced labor fetus or aborted fetus; squares, males; circles, females; rotating square, unknown gender. **(B)** Renal ultrasound of Ⅱ-1 demonstrated bilateral large echogenic kidneys (Right kidney, RK; Left kidney, LK). The red circle indicates the kidney. **(C, D)** Hematoxylin-eosin staining of the renal tissues from the second fetus *postmortem*(Ⅱ-2). **(C)** Photomicrograph shows variably sized cysts (asterisks); **(D)** Photomicrograph (framed zone in c) shows renal tubular cyst (solid arrow) and degeneration and necrosis of glomeruli (dashed arrow).

The family pedigree was shown in Figure 1A. The sonography results of the first fetus (Ⅱ-1) showed enlarged kidneys (left: 51 × 22mm, right: 52 × 29 mm) (Figure 1B) and oligoamnios (amniotic fluid index 0 mm) at 31 weeks of pregnancy. For the second fetus (Ⅱ-2), prenatal ultrasound indicated bilateral normal enhanced fetal kidney echo at 26 weeks, a small amount of amniotic fluid at 23 weeks of pregnancy and the follow-up ultrasound at 26-week gestation showed oligohydramnios (amniotic fluid index 33 mm) and bilateral enlarged echogenic polycystic kidneys. An autopsy of the second fetus was performed, showing the enlarged bilateral kidneys: left kidney 45 × 26 × 22mm, right 35 × 25 × 22 mm, multiple renal tubular cysts, degeneration and necrosis of the bilateral glomeruli (Figure 1C, D). In addition, the size of the liver was 75 × 50 × 30 mm and few visible lobular structures with dilated hepatic sinusoids, massive inflammatory cell infiltration in the intrahepatic vessel walls and fibroplasia was seen.

### Cytocular and molecular genetics examination

**FIGURE 2:**
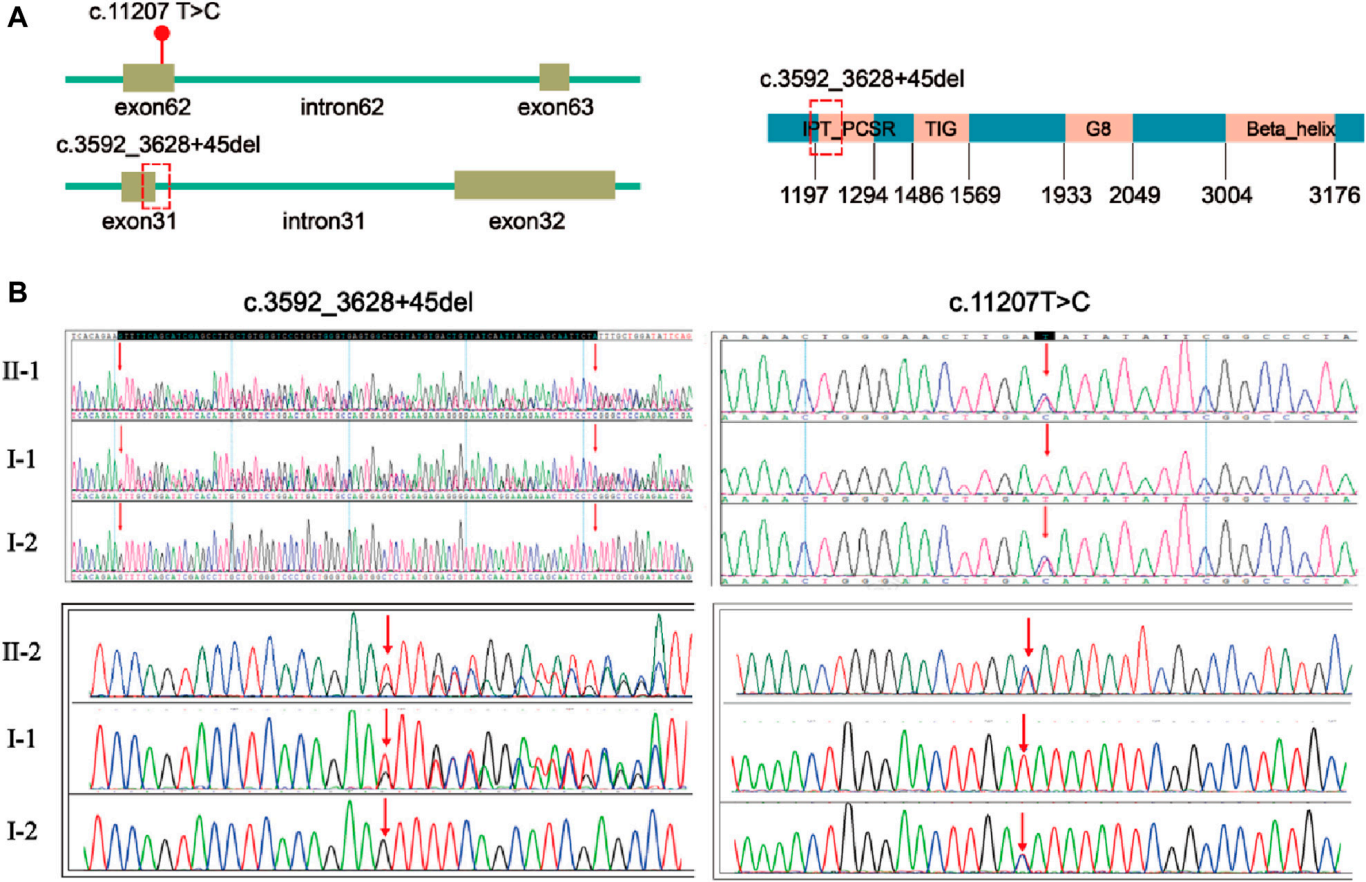
Localization and validation of two compound heterozygous mutations. **(A)** The location of mutation c.11207T>C, p.(Ile3736Thr) and mutation c.3592_3628 + 45del, p? in *PKHD1* gene (NM_138694.4). The red dot indicates the position of mutation c.11207 T>C, p.(Ile3736Thr) in the *PKHD1* gene (NM_138694.4); the red dotted box indicates the position of mutation c.3592_3628 + 45del, p? in the *PKHD1* gene (NM_138694.4) and FPC protein. **(B)** Validation of the variants by Sanger sequencing. The red arrows indicate the variant sites.

Trio-WES of first fetus (II-1) and the couple identified compound heterozygous mutations of the *PKHD1* gene (NM_138694.4) in the first fetus: unreported missense mutation c.11207 T > C (p. Ile3736Thr) in exon 62 and a frameshift deletion c.3592_3628 + 45del, p? between exon 31 and intron 31 (Figure 2A). The mutation c.3592_3628 + 45del is located in the IPT_PCSR domain of FPC protein (Figure 2A). Sanger sequencing result showed that the two mutations were inherited from the mother and the father (Figure 2B). Both the karyotyping and CNV-seq results of the second fetus were normal. However, sanger sequencing verified that the second fetus had the same mutations in the *PKHD1* gene as the first fetus (Figure 2B).

### Splicing analysis of *PKHD1* minigene

**FIGURE 3:**
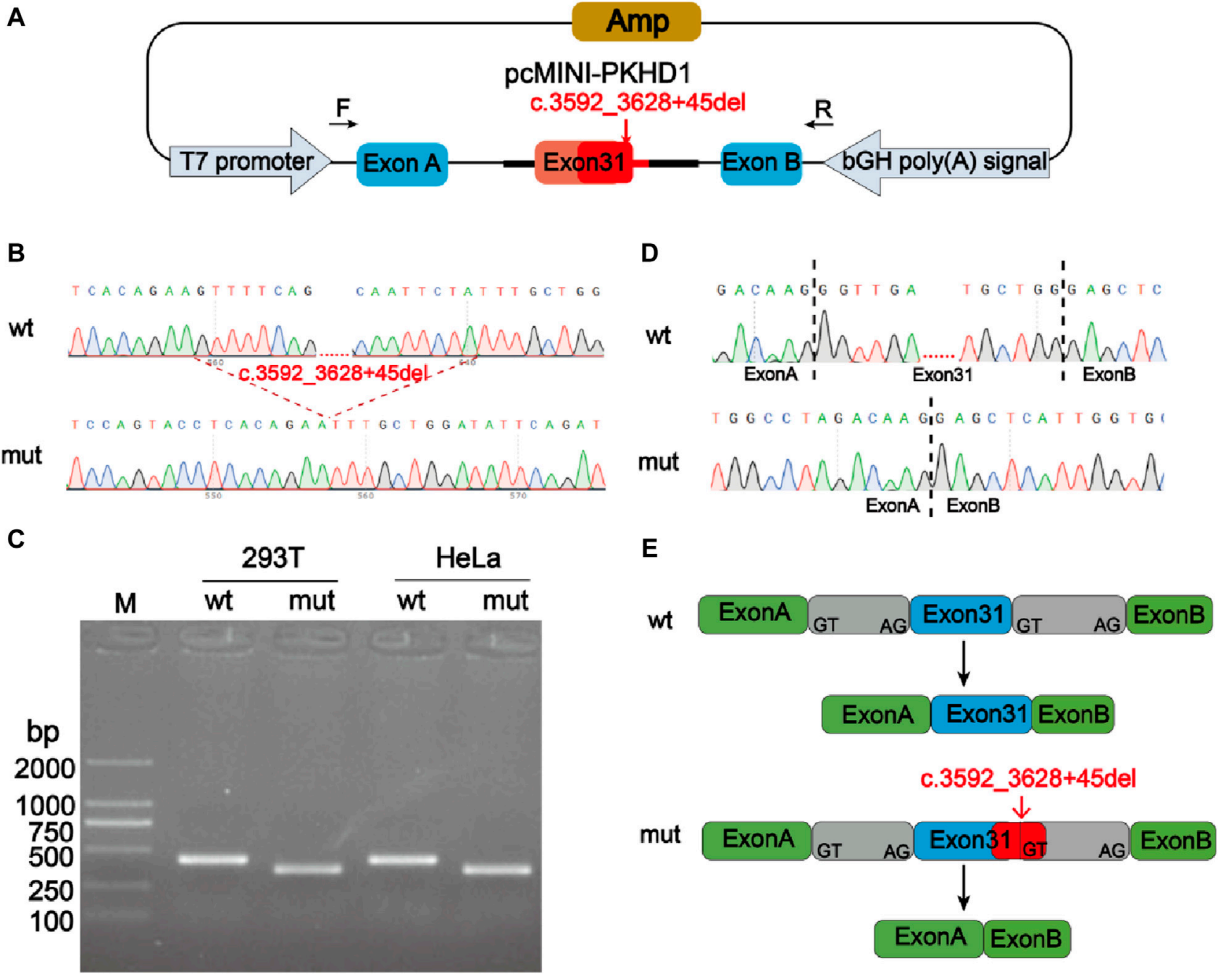
Splicing alteration was identified by a minigene assay using a pcMINI vector. **(A)** Schematic diagram of the minigene and vector construction. **(B)** Sequencing results in the recombinant vector. The top of **(B)** indicates the results of wt minigene sequencing, and the bottom shows the sequencing of the mut (c.3592_3628 + 45del, p?) minigene. The red dotted line indicates the change of mutation. **(C)** Reverse-transcription polymerase chain reaction (RT-PCR) products were separated by electrophoresis of the pcMINI-*PKHD1*-wt/mut vector in 293T and HeLa cells. The different splicing products for wild-type (wt lane, 418 bp) and mutant-type (mut lane, 350bp) were shown on 2% agarose gel electrophoresis and represented graphically. **(D)** The sequencing results for the bands. **(E)** Schematic representation of normal and incorrect splicing of the wt and c.3592_3628 + 45del, p? mutant minigene.

**FIGURE 4:**
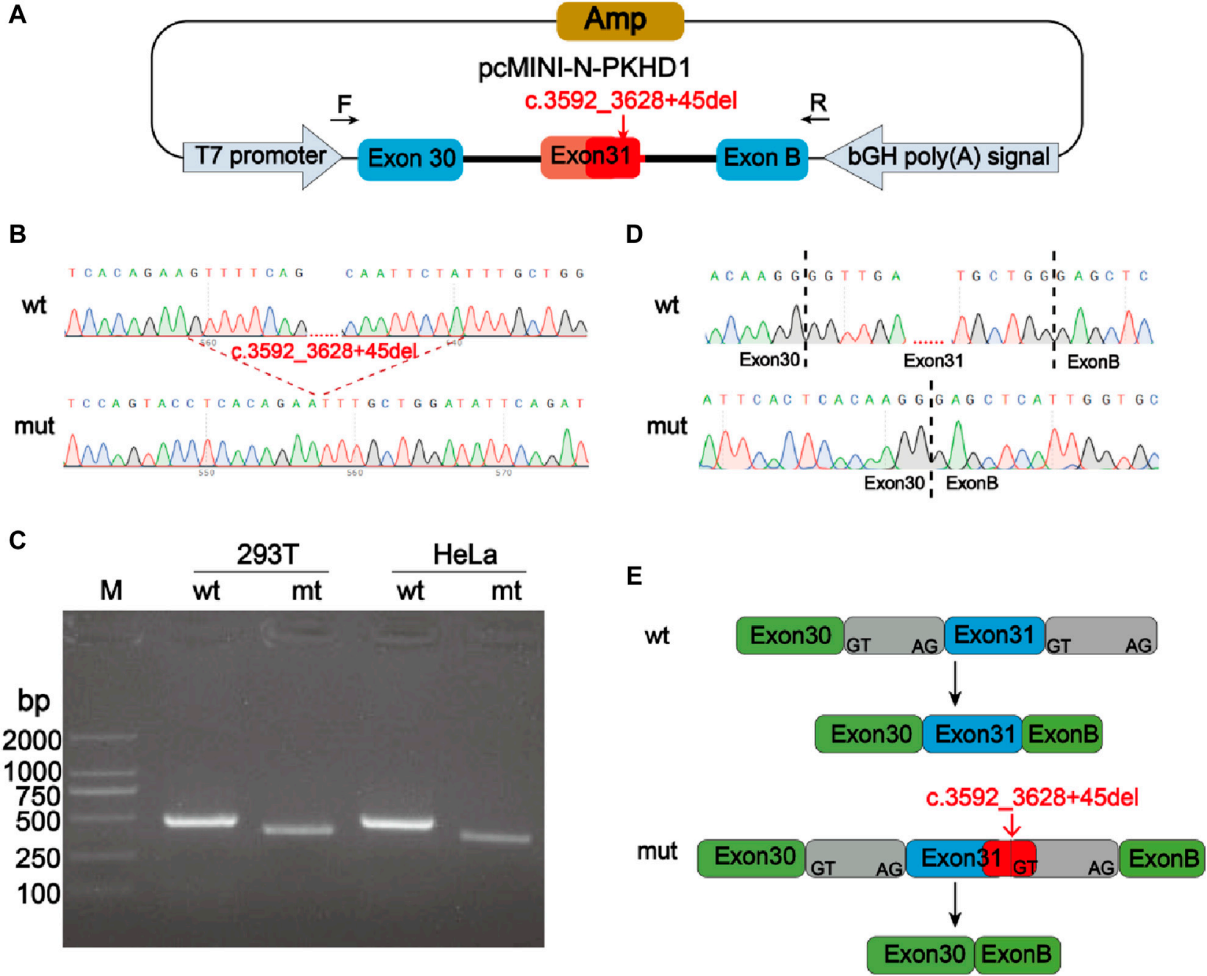
Splicing alteration was identified by a minigene assay using a pcMINI-N vector. **(A)** Schematic diagram of the minigene and vector construction. **(B)** Sequencing results in the recombinant vector. The top of **(B)** indicates the results of wt minigene sequencing, and the bottom shows the sequencing of the mut (c.3592_3628 + 45del, p?) minigene. Both are partial sequencing results. The red dotted line indicates the change of mutation. **(C)** RT-PCR products were separated by electrophoresis of the pcMINI-N-*PKHD1*-wt/mut vector in 293T and HeLa cells. The different splicing products for wild-type (wt lane, 467 bp) and mutant-type (mut lane, 399bp) were shown on 2% agarose gel electrophoresis and represented graphically. **(D)** The sequencing results for the bands. **(E)** Schematic representation of normal and incorrect splicing of the wt and c.3592_3628 + 45del, p? mutant minigene.

Minigene experiment was performed to study the effects of the mutation c.3592_3628 + 45del, p? on the splicing of the *PKHD1* transcript (NM_138694.4) using two groups of recombinant vectors (Figure 3A; Figure 4A). The monoclonal colonies transformed with recombinant vector were used to extract DNA for PCR and verification by sequencing; the fragments were consistent with the expected fragments, confirming that the target fragment, the *PKHD1*-wt/mut minigene, was successfully inserted into the vector. The sequencing results were shown in Figure 3B, Figure 4B. Reverse-transcription polymerase chain reaction (RT-PCR) of total RNA obtained from HeLa and 293T cells, which were transfected with pcMINI-*PKHD1*-wt/mut and pcMINI-N-*PKHD1*-wt/mut. The agarose gel showed that the bands produced by mut were smaller than those produced by wt (Figure 3C; Figure 4C). The size of the pcMINI-*PKHD1*-wt band was 418 bp, which was consistent with the expected size (Figure 3C). DNA sequencing results indicated that the wt minigene formed a normal mRNA comprising exon A (192 bp), exon 31 (68 bp), and exon B (57 bp) [Figure 3E (wt)]. The c.3592_3628 + 45del, p? mutant minigene (pcMINI-*PKHD1*-mut) caused aberrant splicing, resulting in exon 31 skipping, and the splicing mode was exon A (192 bp) and exon B (57 bp) [Figure 3E (mut)]. The size of the pcMINI-N-*PKHD1*-wt band was 467 bp consistent with the expected size (Figure 4C). Sequencing showed that the wt band was spliced normally, and the splicing mode of the band was exon 30 (196 bp), exon 31 (68 bp) and exon B (57bp) [Figure 4E (wt)]. The c.3592_3628 + 45del, p? mutant minigene (pcMINI-N-*PKHD1*-mut) resulted in exon 31 skipping. The splicing mode of the mut band was exon 30 (196bp) and exon B (57bp) [Figure 4E (mut)]. The sequencing results of the bands were shown in Figure 3D, Figure 4D. These results showed that the *PKHD1*-mut minigenes produced alternative transcripts that were different from those of the *PKHD1*-wt minigenes in the 2 cell types.

### Bioinformatics analysis results

The c.3592_3628 + 45del, p? mutation was located between the intron 31 and exon 31 and belonged to “class I mutation region”, which affects splicing (Cartegni et al., 2002). Splice AI predicted that the wt donor site score would decrease by 0.95 and both NetGene2 and NN Splice predicted that the original donor site would be lost. However, the splicing prediction software programs indicated that the c.11207 T > C, p.(Ile3736Thr) mutation is a polymorphism.

**FIGURE 5:**
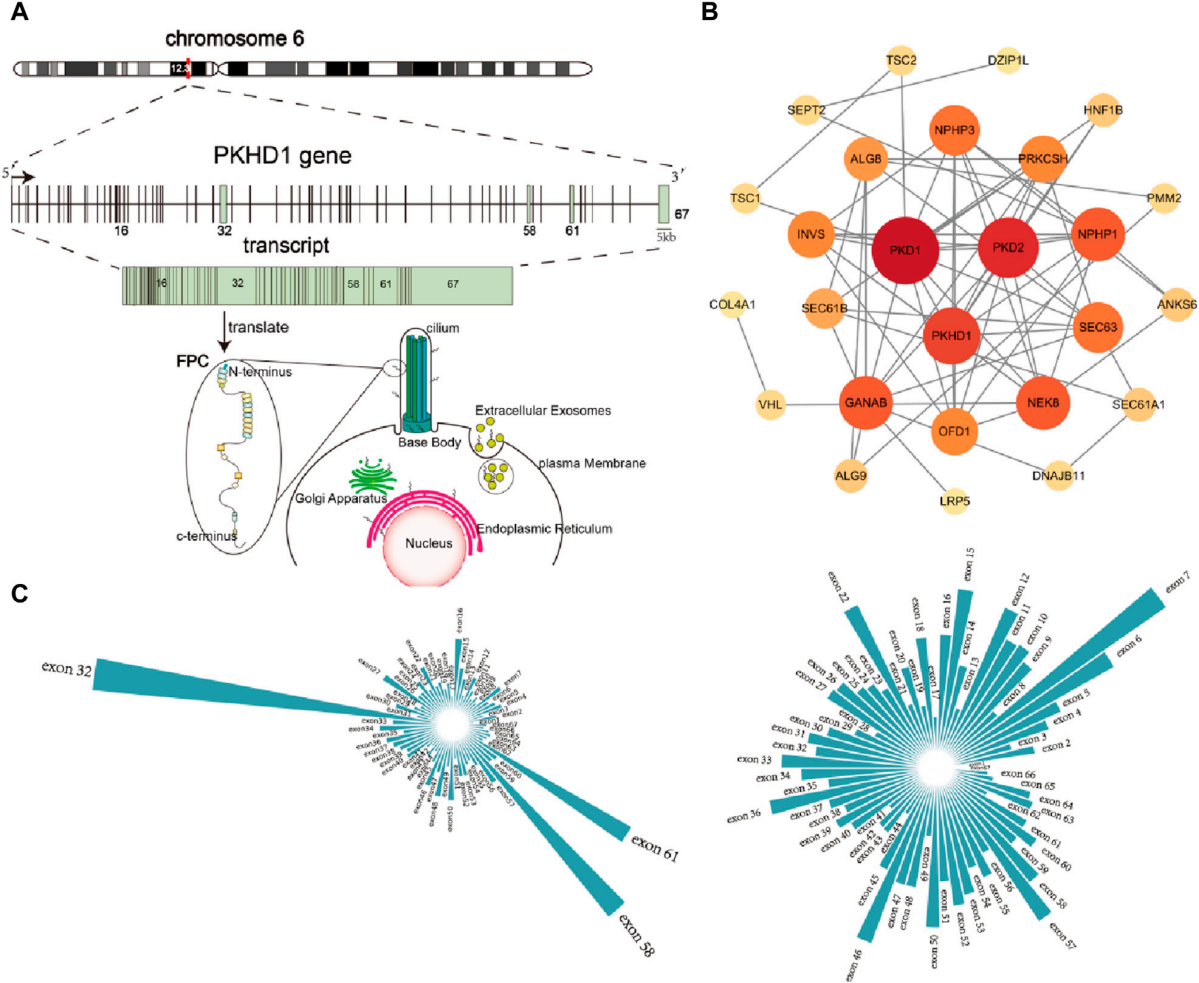
Overview of the *PKHD1* gene **(A)** Chromosome location of *PKHD1* gene, structure of *PKHD1* gene, transcription and translation of *PKHD1* gene, subcellular location of FPC protein. *PKHD1* gene is located on chromosome 6p12.2; The positions of the exons are illustrated and numbered, and the longest transcript is shown from 67 exons for *PKHD1*; localization of FPC protein in cells includes primary cilium, cilium base body, plasma membrane, and Endoplasmic Reticulum, Golgi apparatus, Extracellular Exosomes. **(B)** Protein-protein interaction network of genes with polycystic kidney disease phenotype. The darker the color of the circle or the larger the circle, the more important it is in the network. **(C)** Exon distribution of pathogenic and likely pathogenic mutations in *PKHD1* gene in the Clinvar database and LOVD database.

Protein-protein interaction networks showed that the *PKHD1* gene is a key gene in protein interaction networks by analyzing genes that may cause polycystic kidney phenotype (Figure 5B). And chromosome location of the *PKHD1* gene, the structure of the *PKHD1* gene, transcription and translation of the *PKHD1* gene, subcellular location of the FPC protein were shown in Figure 5A (Menezes et al., 2004; Zhang et al., 2004; Lea et al., 2020). Up to now, it has been found that a large number of mutations in the *PKHD1* gene can cause ARPKD. More than 500 different mutations have been identified in the *PKHD1* gene (Human Gene Mutation Database, HGMD, http://www.hgmd. cf. ac.uk/ac/index.php). The types of variants consist of 396 missense or nonsense, 45 splicing, 81 small deletions, 25 small insertions, 9 small indels, 13 gross deletions, 2 gross insertions or duplications and 1 repeat rearrangement. In addition, we found that more than 1,100 different mutations are reported and the mutations are mainly distributed in the exon 32, 58 and 61 (Figure 5C) by investigating the pathogenic and likely pathogenic *PKHD1* mutations in the Clinvar database (https://www. ncbi. nlm.nih.gov/clinvar) and LOVD3 database (https://databases.lovd.nl/shared/genes/ *PKHD1*). However, there is no significant difference in the ratio of the number of mutations to the length of the exons. Meanwhile, the most common *PKHD1* mutation: c.107 C > T (p.Thr36Met) in exon 3, accounting for approximately 15%–20% of mutated alleles, is a mutational “hotspot” (Hartung and Guay-Woodford, 2014). However, aside from the c.107 T > C (p.Thr36Met) mutation, there is no other evidence of clustering mutations at specific sites in the *PKHD1* gene and most mutations are rare variants spread throughout the gene, many of which are exclusive to a single family (Bergmann, 2017; Burgmaier et al., 2021).

## Discussion

The same two unreported mutations (c.3592_3628 + 45del, p? and c.11207 T > C, p.(Ile3736Thr)) in the fetal *PKHD1* gene (NM_138694.4) were identified inherited from the parents in our study, where related phenotypes of two fetuses showed recurrent enlarged echogenic of polycystic kidney and oligoamnios. The former mutation resulted in the loss of the original donor site, leading to an aberrant splicing product, while the latter has been harmful by biometric software prediction. The above complex heterozygous variations resulted in two similar fetal phenotypic systems of bilateral kidneys.

The mutation c.11207 T > C, p.(Ile3736Thr) in exon 62 was a missense mutation, but at the same site, a mutation T > A leading to another amino acid has been reported in one case to be potentially pathogenic (Van Buren, Neuman, and Sidlow, 2023). This mutation c.11207 T > C led to the change of amino acid 3,638 of FPC from isoleucine to threonine, which may affect the structure and function of FPC. Located 11 amino acids downstream from an N-glycosylation site, the above variants at this locus can adversely affect cell adhesion and communication, thereby hindering terminal differentiation of the epithelial cells that make up renal canaliculi and biliary duct cell adhesion in tandem with other proteins (Onuchic et al., 2002). The c.3592_3628 + 45del variant was a frameshift mutation between exon 31 and intron 31, bringing about changes in the open reading frame and amino acid sequences. It was a classical splicing site, however, its clinical significance remained unknown. Thereafter, it was further confirmed that the variant might have a great impact on splice site function through bioinformatic splice prediction tools. We conducted the minigene experiment to clarify the pathogenicity of the mutation c.3592_3628 + 45del, p? The results *in vitro* showed that the c.3592_3628 + 45del, p? variant caused aberrant splicing, resulting in exon 31 skipping. And the verified results of the pcMINI and pcMINI-N vectors were same. Moreover, the premature termination codon was produced in codon 29 of exon 32, which resulted in a truncated FPC protein with a length of 1214aa. The truncated FPC protein lacked the partial IPT domain, the parallel beta-helix 1 (PbH1) and the TM domain, compared with normal FPC protein, which resulted in loss of FPC protein function.

According to the ACMG guidelines (Richards et al., 2015; Monaghan et al., 2020), the evidence for the c.3092_3628 + 45del, p? variant was “PVS1 + PS3 + PM2 + PP3 + PP4” and the mutation c.3592_3628 + 45del, p? was judged to be a pathogenic variant. Similarly, the evidence for the c.11207 T > C, p.(Ile3736Thr) variant was “PM2 + PM3 + PP3 + PP4” and was judged to be an uncertain significance variant.

The mode of inheritance of polycystic kidney disease caused by the *PKHD1* gene is thought to be recessive, however, in some cases, it has also been found that heterozygous carriers of mutations in the *PKHD1* gene may develop liver and kidney phenotypes. In our study, the parents, as heterozygous carriers of c.11207 T > C, p.(Ile3736Thr) and c.3592_3628 + 45del, p?, respectively, owned normal phenotypes, while both of their fetuses with polycystic kidney and liver phenotypes carried the compound heterozygous mutation of c.11207 T > C, p.(Ile3736Thr) and c.3592_3628 + 45del, p? Therefore, both of the mutations are recessive inheritance and heterozygous carriage does not cause kidney or liver phenotypes. There may be other reasons why heterozygous carriers of *PKHD1* mutations develop liver and kidney phenotypes. Other genes, including those encoding ciliary proteins, may contribute to cyst formation through synergistic heterozygosity, which could explain why only a subset of *PKHD1* mutation carriers develop these liver or kidney findings (Vockley et al., 2000; Gunay-Aygun et al., 2011; Vockley et al., 2019). In addition, some researchers have also suggested another possible genetic model for cyst pathogenesis that the polycystic liver phenotype in *PKHD1* heterozygous carriers could occur through somatic second hit mutations(Besse et al., 2020). However, the two genetic models for cyst pathogenesis in *PKHD1* heterozygous mutation carriers require more clinical information to prove it.

Although at the same site in one case, the mutation leading to the change of another amino acid has been reported to be pathogenic and predicted to be deleterious by bioinformatic methods, we still need *in vitro* functional experiments to further verify the influence of the mutation c.11207 T > C, p.(Ile3736Thr) on FPC protein function. Another limitation is that RNA transcriptomic studies were limited due to the failure of RNA extraction from fetus kidney samples.

In summary, we identified two unreported mutations in the *PKHD1* gene and evaluated the functional significance of the c.3592_3628 + 45del, p? mutation using a minigene splicing assay. The c.3592_3628 + 45del, p? variant was confirmed to be an aberrant splicing, resulting in exon 31 skipping, as a pathogenic site. This novel finding broadens the variant spectrum of the *PKHD1* gene and provides a basis for genetic counseling and diagnosis of ARPKD.

## Data Availability

The data presented in the study are deposited in the figshare repository, accession number “Pathogenic Relationship Between Phenotypes of ARPKD and Novel Compound Heterozygous Mutations of PKHD1”: https://figshare.com/articles/journal_contribution/Pathogenic_Relationgship_Between_Phenotypes_of_ARPKD_and_Novel_Compound_Heterozygous_Mutations_of_PKHD1/26074243.
