# Supplementary material for: Pathogenic relationship between phenotypes of ARPKD and novel compound heterozygous mutations of PKHD1
Source: Front Genet. 2024 Jul 2;15:1429336. doi: 10.3389/fgene.2024.1429336 (PMC11250243; doi:10.3389/fgene.2024.1429336)
Supplement: Supplementary file 1 [file Table1.DOCX]

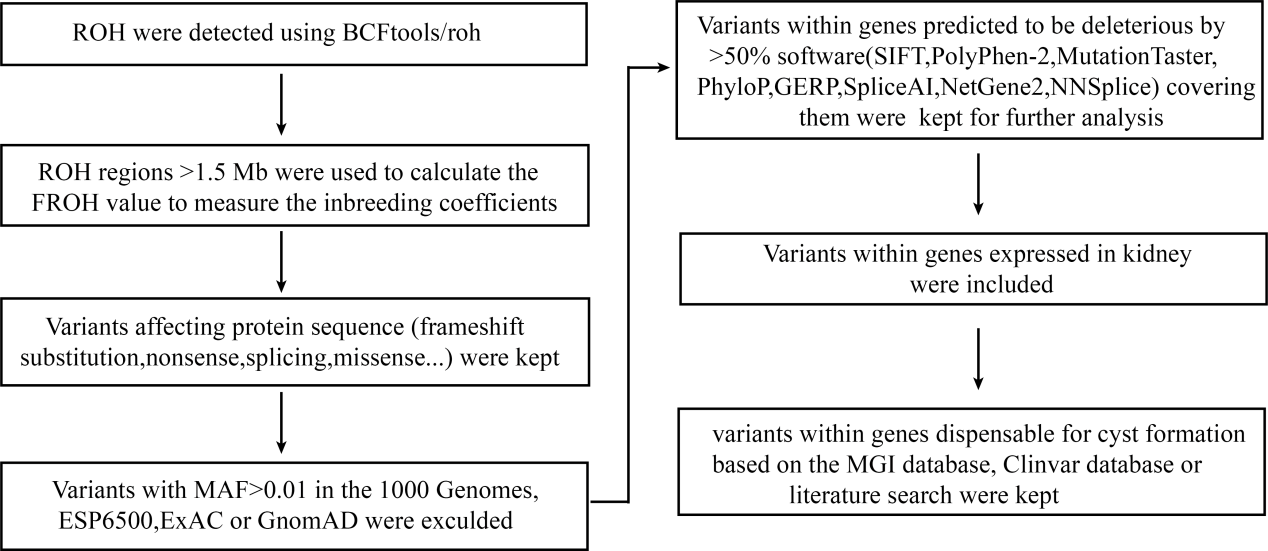


**Figure S1.** The pipeline for whole-exome sequencing data filtration analysis of the Chinese family. Runs of homozygosity (RoH) were first detected using BCFtools/RoH and RoH regions >1.5 Mb were used to calculate the FROH value to measure the inbreeding coefficients using our inhouse scripts. The first fetus was found to have a FROH <0.01 and was thought to be offspring of nonconsanguineous marriages. Afterwards, variants meeting the following conditions were given preference: (1) variants potentially affecting protein sequence (nonsense, missense, splice-site variants, and coding indels); (2) variants with minor allele frequencies (MAF) <0.01 in 1000 Genomes project, ESP6500, or GnomAD database; (3) loss-of-function variants or potentially deleterious missense variants predicted by 7 software programs including Sorting Intolerant From Tolerant (SIFT), PolyPhen-2, MutationTaster, MutationAssessor, FATHMM, GERP++, and SiPhy for predicting the pathogenicity of variants. Variants predicted to be deleterious by at least half of the programs covering the variants were kept for further analysis; (4) Variants within genes expressed in kidney. Finally, variants within genes causing renal cyst based on the MGI database, Clinvar database or literature search were kept.

**Table S1** Primers for the construction of pcMINI and pcMINI-N vector

| Primers | Sequences (5′ to 3′) |
| --- | --- |
| 55982-F | gtggtgctctttgctggtta |
| 56276-F | tagctgggtgtggtggcaca |
| 58465-R | cccagaagccaccactttaa |
| 58673-R | gcacctgtggaagtcctagg |
| pcMINI-N-PKHD1-Kpnl-F | GCTTGGTACCATGGCGGTGAGACCCTGGTCATT |
| pcMINI-N-PKHD1-Xhol-R | tttcCTCGAGtccttgcatgcacagcatct |
| pcMINI-PKHD1-Kpnl-F | ggtaGGTACCctagcattcagctaggtaat |
| pcMINI-PKHD1-Xhol-R | tttcCTCGAGacatgacccacagcataacc |
| PKHD1-mut-F | CAGTACCTCACAGAAtttgctggatattca |
| PKHD1-mut-R | tgaatatccagcaaaTTCTGTGAGGTACTG |

Note:F,forward;R,reverse;55982-F,58673-R is a set of primers;56276-F,58465-R is a set of primers.

Both wild-type and mutant-type target DNA fragments from normal human genomic DNA were cloned using nested PCR methods with PrimerSTAR MAX DNA Polymerase Kit (TaKaRa, R045A, China) according to the manufacturer’s instructions. Primers used for cloning in the minigene assay are listed in Table S1. The PCR products, pcMINI-PKHD1-wt/pcMINI-PKHD1-mut and pcMINI-N-PKHD1-wt/pcMINI-N-PKHD1-mut were purified and inserted into the eukaryotic expression vector pcMINI and pcMINI-N using KpnI-F/XhoI to construct two sets of plasmids: pcMINI-PKHD1-wt/mut and pcMINI-N-PKHD1-wt/mut.

**Table S2** PCR amplification primers

| Primers | Sequences (5′ to 3′) |
| --- | --- |
| pcMINI-F | CTAGAGAACCCACTGCTTAC |
| pcMINI-R | GCCCTCTAGActggtcattccggctc |
| pcMINI-N-F | CTAGAGAACCCACTGCTTAC |
| pcMINI-N-R | TAGAAGGCACAGTCGAGG |

Note:F,forward;R,reverse.

For pcMINI-PKHD1-wt and pcMINI-PKHD1-mt, the cDNA was used for PCR amplification with pcMINI-F/pcMINI-R as the primers, and PCR was performed using the cDNA from pcMINI-N-PKHD1-wt and pcMINI-N-PKHD1-mt with pcMINI-N-F/pcMINI-N-R as the primers.
